# Is it time to consider visual feedback systems the gold standard for chest compression skill acquisition?

**DOI:** 10.1186/s13054-017-1740-z

**Published:** 2017-07-04

**Authors:** Andrea Cortegiani, Vincenzo Russotto, Enrico Baldi, Enrico Contri, Santi Maurizio Raineri, Antonino Giarratano

**Affiliations:** 1 Department of Biopathology and Medical Biotechnologies (DIBIMED), Section of Anaesthesia, Analgesia, Intensive Care and Emergency, University Hospital Paolo Giaccone, University of Palermo, Via del Vespro 129, 90127 Palermo, Italy; 2 Pavia nel Cuore, Via de Canistris 7, 27100 Pavia, Italy; 3 School of Cardiovascular Disease, University of Pavia, Viale Golgi 19, 27100 Pavia, Italy; 4 School of Anesthesia and Intensive Care, University of Pavia, Viale Golgi 19, 27100 Pavia, Italy

**Keywords:** Cardiac arrest, Cardiopulmonary resuscitation, Chest compressions

High-quality chest compressions are pivotal for improving survival from cardiac arrest. The rate and depth of compressions, chest recoil and hand position are important parameters affecting the overall quality of chest compressions, which is correlated with blood flow and oxygen delivery to the heart and brain and, consequently, with rate of ROSC and neurologically intact survival at hospital discharge [1]. During the last decade, some automated feedback devices have been investigated to improve CPR performance during cardiac arrest [2]. However, the applicability of these systems on a large scale is questionable and more attention has been focused on FS for training with unclear effects [3].

Two recently published RCTs brought new high-quality evidence on this topic. Both RCTs evaluated the effect of an automated computerized real-time FS (Laerdal QCPR®) able to measure CPR quality, which can be connected wirelessly to a training mannequin and displayed on pads or laptops (Additional file 1).

Baldi et al. [4] randomized 450 laypersons of various age participating in BLS courses in a three-arm study. The authors demonstrated that both a 1-minute training or a 10-minute training with the FS was superior to a standard course in terms of the percentage of compressions with correct depth, with complete chest recoil and with correct hand position. In this trial, assessment of the chest compression skill acquisition was performed at the end of the course.

Cortegiani et al. [5] randomized 144 trainers in a two-arm study comparing a standard course plus a 2-minute chest compressions training with the FS versus instructor-based feedback only. The intervention group demonstrated a significantly higher overall quality and percentage of correctly released chest compressions and a more appropriate compression rate. Interestingly, in this trial, outcomes assessment was performed 7 days after the course. The median age of participants was lower than for the other trial (17 years for both groups) because it specifically focused on secondary school students.

There is now high-grade evidence to support the effect of a visual FS in terms of chest compression skill acquisition for laypersons. Further research should evaluate the effect of a visual FS at longer time points and for training (and retraining) of healthcare personnel, focusing on patient-centered outcomes. Moreover, high-quality studies comparing different FSs are needed.

## Additional file

Additional file 1: Shows the graphic interface of the Laerdal QCPR® feedback system. Screenshot representing how Laerdal QCPR® provides real-time visual feedback during training. In this case, compressions are too shallow, 40 mm for the last one, with incomplete chest recoil (another yellow arrow suggests you should allow complete chest recoil), and the compression rate is too low, 84 compressions/minute (a continuous yellow line shows that the compressions are not in the correct range). The system recognizes as correct parameters those recommended by international guidelines. (TIFF 123 kb)

## Abbreviations

BLS: Basic life support
CPR: Cardiopulmonary resuscitation
FS: Feedback system
RCT: Randomized controlled trial
ROSC: Return of spontaneous circulation
