# Supplementary figures and images for: Is it time to consider visual feedback systems the gold standard for chest compression skill acquisition?
Source: Crit Care. 2017 Jul 4;21:166. doi: 10.1186/s13054-017-1740-z (PMC5496175; doi:10.1186/s13054-017-1740-z)

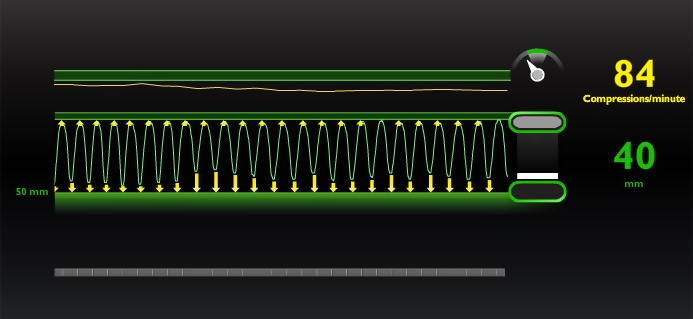

Supplement: Additional file 1: — Shows the graphic interface of the Laerdal QCPR® feedback system. Screenshot representing how Laerdal QCPR® provides real-time visual feedback during training. In this case, compressions are too shallow, 40 mm for the last one, with incomplete chest recoil (another yellow arrow suggests you should allow complete chest recoil), and the compression rate is too low, 84 compressions/minute (a continuous yellow line shows that the compressions are not in the correct range). The system recognizes as correct parameters those recommended by international guidelines. (TIFF 123 kb) [file 13054_2017_1740_MOESM1_ESM.tiff]
